# Bilateral Patella Dislocation after Total Knee Arthroplasty: A Report of Two Cases and a Review of the Literature

**DOI:** 10.7759/cureus.1075

**Published:** 2017-03-04

**Authors:** Raju Vaishya, Amit Kumar Agarwal, Sagar Panthi, Vipul Vijay, Abhishek Vaish

**Affiliations:** 1 Orthopaedics, Indraprastha Apollo Hospitals; 2 Orthopaedics Department, Nepal Medical College

**Keywords:** patellar dislocation, total knee arthroplasty, complications, maltracking, surgery

## Abstract

Patellar instability is a known but catastrophic complication after total knee arthroplasty (TKA). The occurrence of bilateral dislocation of the patella after TKA is exceedingly rare. It may present as anterior knee pain, and diagnosis can easily be made clinically or by plain radiographs. Early diagnosis with surgical realignment and repair of the extensor mechanism can provide good outcomes after this complication.

## Introduction

Postoperative complications may impair the outcome of total knee arthroplasty (TKA). Patellar instability is one of the causes for revision surgery [1]. It may occur after TKA with or without patellar resurfacing. Subluxation is more common than dislocation, but the incidence of symptomatic instability leading to revision is few (0.5 to 0.8%) [2]. The etiology of patellofemoral instability can be related to (a) the surgical technique and component positioning, (b) extensor mechanism imbalance, and (c) other causes. Component malposition during surgery is one of the most common causes of patellar instability [3]. Placing the components in internal rotation in the transverse plane increases the Q angle, leading into lateral patellar maltracking [4]. We describe our experience of two cases with postoperative simultaneous bilateral patellar dislocation after bilateral TKA. 

## Case presentation

Informed patient consent was obtained for treatment from both patients.

### Case 1

**Figure 1:**
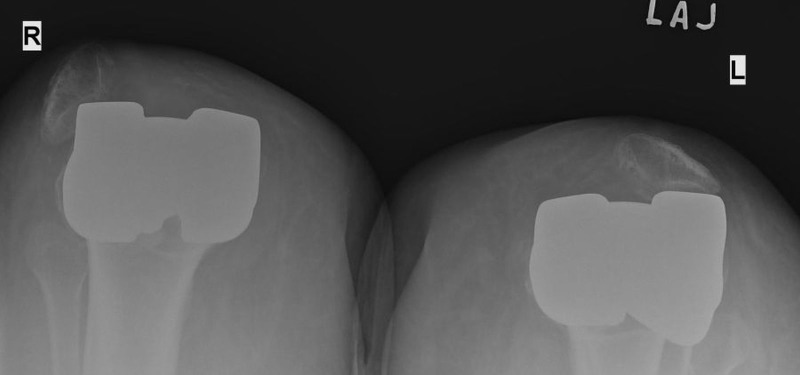
Skyline x-ray view of both knees showing the bilateral dislocation of the patellas.

A 55-year-old woman presented with bilateral anterior knee pain nine months after a simultaneous bilateral TKA, using posterior stabilized Scorpio NRG™ prosthesis (Stryker, Kalamazoo, MI) without patellar resurfacing. The pain started following forceful bending of both knees after a fall at home on a slippery floor. Right knee passive range of motion (ROM) was 20-105 degrees and left knee passive ROM was 15-100 degrees. Plain radiographs of both knees revealed bilateral dislocation of both patellas (Figure 1).

**Figure 2:**
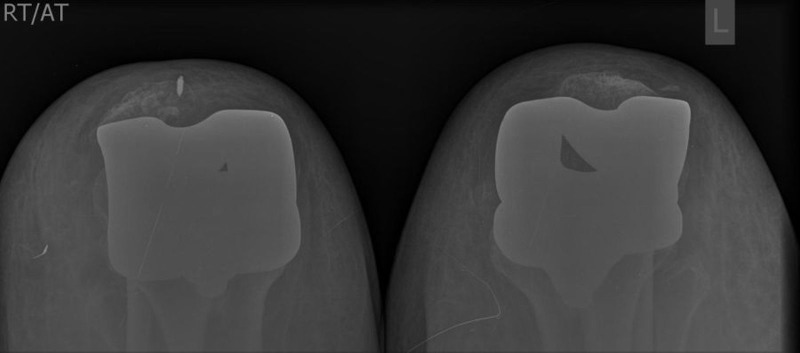
Postoperative follow-up x-ray with skyline view both knees showing normal position of the patellas.

The patient underwent simultaneous bilateral extensor mechanism repair. Postoperative x-ray was satisfactory (Figure 2). Following surgery, the patient was placed in a knee immobilizer for six weeks with restricted ROM and non-weight-bearing. After six weeks, she was allowed weight-bearing as tolerated in a knee brace, eventually progressing to unrestricted ROM. The brace was discontinued at 12 weeks. At 18 months follow-up, both the knees remained stable with no other complaints. The active ROM was 0-115 degrees in right and 0-110 degrees in left knee at the last follow-up.

### Case 2

**Figure 3:**
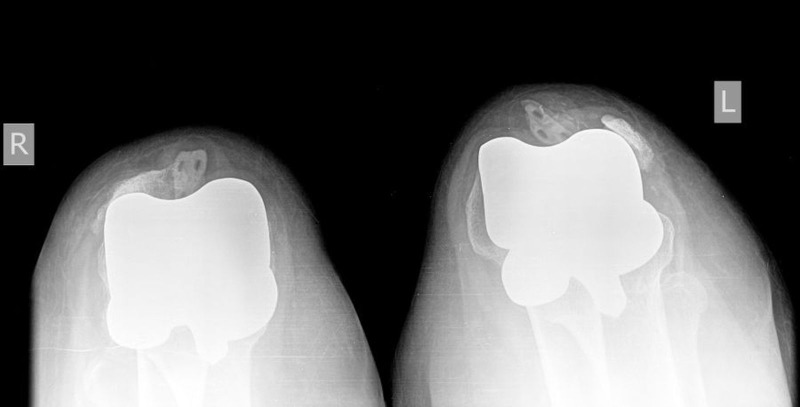
X-ray with skyline view of both knees showing component loosening, along with dislocation of the patella

A 57-year-old woman presented with the acute onset of bilateral knee pain following a fall from standing height. She had simultaneous bilateral TKAs seven months previously using posterior stabilized Triathalon™ prostheses (Stryker, Kalamazoo, MI) with patellar resurfacing. Medical comorbidities included morbid obesity (BMI: 58.9 kg/m2), hypertension, and Type II diabetes mellitus. Passive ROM was 15-100 degrees in both knees, and radiographs revealed bilateral patellar dislocations with component dissociation (Figure 3). 

The patient underwent simultaneous bilateral patelloplasties with the removal of the patellar components and extensor mechanism repair. Postoperatively, the patient was placed in a knee immobilizer for six weeks with limited ROM and weight-bearing. After six weeks, she was allowed weight-bearing as tolerated in a knee brace, eventually progressing to unrestricted ROM. The brace was discontinued at 12 weeks. At her 14-month follow-up, she remained pain-free with active ROM of 10-100 degrees in both knees.

## Discussion

As per literature, the reported incidence of patellar problems after TKA has ranged from 5% - 30% in various studies. However, patellar dislocation is infrequent but can cause disabling symptoms [5]. In a study in 2003, Rand reported complications of extensor mechanism were the main cause for revision seen in 12% of their cases [6]. Symptoms of patellar instability are anterior knee pain during stressful activities, such as stair climbing or rising from a chair. The sudden onset of a non-symptomatic period is more likely to be related to component failure or extensor mechanism disruption. Knee pain may or may not be associated in patients presenting with a giving way or a buckling sensation of the knee. The range of motion is decreased and the patient is unable to do full flexion of the knee. On palpation, there is tenderness over extensor mechanism. While performing ROM, dislocation or subluxation can be elicited.

Patellar component malposition usually reflects a technical error in cutting the patella. The effort is to make a symmetrical hockey-puck shaped structure that has an equal thickness from top to bottom and from side to side. Under-resection of the lateral facet or the distal pole will lead to the tightness of the lateral retinaculum and a tendency to subluxate laterally. Grace and Rand were clearly able to show that increasing the patellar thickness or stuffing the joint could lead to lateral patellar instability [7].

Several authors have noted the potential of a valgus knee to predispose to patellar instability. Not uncommonly, these knees have a chronically subluxated or dislocated patella. Often, there is lateral retinacular tightness associated with the longstanding deformity and, if not addressed with a release, can cause subluxation. The lateral condyle in a valgus deformity may be smaller than normal in dimension. With the use of appropriate instrumentation, especially intramedullary femoral guides, the chance of creating a postoperative valgus deformity is decreased; this problem may be even further reduced with the advent of computer-assisted navigation of alignment that has been shown to be even more precise.

Lateral retinacular tightness remains a subtle cause of patellar instability but usually does not result in a clinical problem by itself. Some authors have clearly shown that surgical technique, such as basing the femoral resection on the posterior condylar axis, creates femoral internal rotation which will lead to the need for lateral release. Therefore, if a patient has patellar instability, the surgeon must look for other causes or problems than the simply tightness of the lateral retinaculum.  A chronically dislocated or subluxated patella is the exception, however, and retinacular tightness can be the primary source in this case. Unfortunately, these cases may also have medial retinacular weakness or atrophy, and every attention to the details of reconstruction is needed.

The common causes of patellar instability include the weakness of the medial retinaculum after total knee arthroplasty, expanding hematoma, inadequate surgical closure, over intensive physical therapy, or injury. Rare causes of patellar instability have resulted from surgical misadventure, such as placing the right femoral component in the left knee and placing a ridged anatomical patella component so that the ridge was parallel to the transverse joint plane and not vertical. 

Bilateral patellar instability is an uncommon sequela of TKA. Surgical technique is probably the most important cause of this problem due to abnormal component rotation or limb malalignment. With evolutionary methods of technique and prosthetic design, instability now has become a rare incidental finding after TKA. Component malposition must be treated with component revision while proximal soft tissue realignment should manage soft tissue imbalance. Distal tibial tubercle transfer may be utilized but must be done with great care to avoid complications of skin necrosis or patellar ligament disruption.

Problems arising from the patellofemoral joint are still one of the leading causes of disability in TKA. Recent publications show that retention of the articular surface of the patella leads to increased incidence of anterior knee pain and the subsequent need for patellar resurfacing. However, patellar resurfacing may potentially result in other complications, and if the principles are not followed, then it leads to patellofemoral instability, patellar fracture, component loosening or dissociation, avascular necrosis, patellar clunk, or tendon rupture being among the potential complications [8]. Loosening or dissociation of the patellar component is quite uncommon. Osteoporotic periprosthetic bone, poor cementing or fixation techniques, femoral, tibial, or patellar component malpositioning, eccentric tracking compromise in patellar vascularity with the lateral release, infrapatellar fat pad excision during the primary surgery, trauma, and excessive body weight are factors contributing to that result. Additionally, certain metal-backed designs that have been banned from the market can also be associated with such kinds of complications [9].

The diagnosis of patella component dissociation can be diagnosed by detailed history and plain radiographs, including a skyline view. The management of this disorder includes either component removal and observation or revision of the arthroplasty. Before embarking on any decision, the physician should consider certain factors: the etiologic contributing factors, a local condition in the patellar articular surface after component dissociation, and patient parameters, including age, body weight, suitability for surgery, and patient cooperation. Few authors from their studies have concluded that patellar maltracking is because of probable inadequate external rotation of the femoral component and repetitive trauma has resulted in patellar component dissociation. Our patient’s assessment and treatment was kept simple using arthroscopic surgery. Removing the component alone has led to considerable improvements in her symptoms.

Dussa and Singhal [10] reported that extensor disruptions of the knee following the TKA are uncommon. They described a case of postoperative bilateral simultaneous extensor mechanism disruption following simultaneous bilateral TKA. Their patient sustained open wounds of both the knees and the extensor mechanism was repaired successfully on both sides, but the outcome was less than satisfactory.

## Conclusions

The patellar dislocation following the total knee arthroplasty (TKA) is a disabling condition. We have described two cases of bilateral simultaneous patellar dislocation following TKA. Early diagnosis with surgical realignment and repair of the extensor mechanism can provide good outcomes after this complication.

## References

[1] Malo M, Vince KG (2003). The unstable patella after total knee arthroplasty: etiology, prevention, and management. J Am Acad Orthop Surg.

[2] Rand JA (1994). The patellofemoral joint in total knee arthroplasty. J Bone Joint Surg Am.

[3] Akagi M, Matsusue Y, Mata T, Asada Y, Horiguchi M, Iida H, Nakamura T (1999). Effect of rotational alignment on patellar tracking in total knee arthroplasty. Clin Orthop Relat Res.

[4] Eisenhuth SA, Saleh KJ, Cui Q, Clark CR, Brown TE (2006). Patellofemoral instability after total knee arthroplasty. Clin Orthop Relat Res.

[5] Merkow RL, Soudry M, Insall JN (1985). Patellar dislocation following total knee replacement. J Bone Joint Surg Am.

[6] Rand JA (2003). Extensor mechanism complications following total knee arthroplasty. J Knee Surg.

[7] Grace JN, Rand JA (1988). Patellar instability after total knee arthroplasty. Clin. Orthop.

[8] Schiavone Panni A, Cerciello S, Del Regno C, Felici A, Vasso M (2014). Patellar resurfacing complications in total knee arthroplasty. Int Orthop.

[9] Bayley JC, Scott RD, Ewald FC, Holmes GB Jr (1988). Failure of the metal-backed patellar component after total knee replacement. J Bone Joint Surg Am.

[10] Dussa CU, Singhal K (2005). Bilateral simultaneous extensor mechanism disruption following simultaneous bilateral total knee replacement. Arch Orthop Trauma Surg.

